# LncRNA MNX1-AS1 promotes ovarian cancer process via targeting the miR-744-5p/SOX12 axis

**DOI:** 10.1186/s13048-021-00910-0

**Published:** 2021-11-17

**Authors:** Yang Shen, Mengmeng Lv, Yichen Fang, Jin Lu, Yuzhong Wu

**Affiliations:** Department of Gynecologic Oncology, Jiangsu Cancer Hospital & Jiangsu Institute of Cancer Research & The Affiliated Cancer Hospital of Nanjing Medical University, 42 Baiziting Street, Nanjing, 210009 Jiangsu China

**Keywords:** Ovarian cancer, Long non-coding RNA, MNX1-AS1, MiR-744-5p, SOX12

## Abstract

**Purpose:**

Ovarian cancer (OC) is the most common malignancy in women with high mortality. Increasing studies have revealed that long non-coding RNA (lncRNA) MNX1-AS1 has a promoting effect on various cancers. However, the mechanisms of MNX1-AS1 in OC are still unclear. Therefore, this study focused on exploring the mechanisms of MNX1-AS1 in OC.

**Materials and methods:**

The expression of SOX12 at the protein level was detected by western blot. Cell proliferation was detected by CCK8 assay and colony formation assay. Cell cycle and cell apoptosis were detected by flow cytometry. Wound-healing assay, transwell assay and western blot were used to detect the ability of cell migration and invasion. The target binding was confirmed through the luciferase reporter assay.

**Results:**

The expression of MNX1-AS1 was increased in OC tumor tissues and cells. Elevated MNX1-AS1 expression is associated with advanced stage and lower overall survival rate. Knockdown of MNX1-AS1 inhibited cell proliferation, migration and invasion, blocked cell cycle, and promoted cell apoptosis in SKOV-3 and OVCAR-3 cells. MNX1-AS1 was competitively binding with miR-744-5p, and its downstream target gene was SOX12. miR-544-5p expression was decreased, while SOX12 expression was increased in OC tumor tissues and cells. Overexpression of miR-744-5p inhibited cell proliferation, migration, invasion and promoted cell apoptosis in SKOV-3 and OVCAR-3 cells.

**Conclusion:**

MNX1-AS1 promoted the development of OC through miR-744-5p/SOX12 axis. This study revealed a novel mechanism of MNX1-AS1 in OC, which may provide a new treatment or scanning target for OC.

**Supplementary Information:**

The online version contains supplementary material available at 10.1186/s13048-021-00910-0.

## Introduction

Ovarian cancer (OC) is the most common malignancy in women [1]. More than 70% of OC patients are diagnosed at advanced stages (III and IV stage) due to early non-specific symptoms and a lack of effective screening methods [2]. It is the main reason the 5-year survival rate of patients with OC is still only 20–40%, although the treatment methods such as surgery, radiotherapy and chemotherapy have improved [3]. In addition, a poor understanding of the pathogenesis of OC also contributes to high mortality [4]. Therefore, exploring the molecular mechanisms of OC is necessary for developing novel therapeutic methods and screening targets.

Long non-coding RNAs (lncRNAs) are the essential types of non-coding RNAs involved in the occurrence and development of various cancers [5, 6]. A large number of studies have shown that lncRNAs function as competitive endogenous RNAs (ceRNAs) by competitively occupying the binding sequences of miRNAs, then regulating the expression of downstream target genes [7]. For example, the level of lncRNA-CDC6 is elevated in breast cancer, and it promotes cell proliferation and migration through sponging of miR-215 and further regulating the expression of CDC6 [8]. On the other hand, LncRNA MT1JP expression is decreased in gastric cancer, and it inhibits the process of gastric cancer through competitively binding to miR-92a-3p and regulating the expression of FBXW7 [9].

In addition, increasing studies reported that abnormal expression of lncRNAs involved in the process of cancer, including cell proliferation, cell migration, cell apoptosis, epithelial-mesenchymal transition (EMT), cell resistance [10–12]. Previous reports have verified that several lncRNAs are involved in the development of OC. For instance, Lin et al. have found that lncRNA DANCR expression is increased in OC tissues and cells. Furthermore, their study confirmed that DANCR promotes tumor growth and angiogenesis through targeting miR-145 and then regulating the expression of VEGF [13].

LncRNA MNX1-AS1 is an antisense RNA of motor neuron and pancreas homeobox protein 1 (MNX1) gene. It has been reported that MNX1-AS1, as an oncogene, promotes the development of many cancers, including intrahepatic cholangiocarcinoma, cervical cancer, lung cancer and hepatocellular carcinoma [14–17]. In OC, Li et al. confirmed that MNX1-AS1 expression is increased in tumor tissues than matched normal tissues, and overexpression of MNX1-AS1 is associated with poor clinical outcomes [18]. Additionally, Yan et al. found that knockdown of MNX1-AS1 inhibits cell proliferation and migration in OC [19]. Nevertheless, the mechanism of MNX1-AS1 in OC has not been fully elucidated. Hence, this study focused on exploring the underlying mechanism of MNX1-AS1 in OC.

Along with the development of RNA studies, it has been found that mutations or aberrant expression of microRNAs (miRNAs) are closely associated with the development of cancers such as OC [20], and miRNAs as oncogenes or anticancer genes have increasingly attracted attention from researchers. For example, MiR-744-5p is differentially expressed in various cancers and participates in tumorigenesis [21]. Furthermore, MiR-744-5p is underexpressed in many cancers such as colorectal cancer, cervical cancer, and glioblastoma. Moreover, overexpression of miR-744-5p can inhibit cell proliferation, migration, and invasion [22–24]. Additionally, miR-744-5p also downregulates epithelial ovarian cancer cell expression, and its expression can induce apoptosis [21]. However, the mechanism of miR-744-5p on OC is not fully defined and still requires further investigation.

In this study, we confirmed that MNX1-AS1 expression was increased in OC tumor tissues and cells. Furthermore, our results confirmed that MNX1-AS1 promoted the development of OC through miR-744-5p/SOX12 pathway. Thus, this study revealed a novel mechanism of MNX1-AS1 in OC, which may provide a new treatment or scanning target for OC.

## Materials and methods

### Clinical tissues sample

OC tumor tissues (*n* = 30) and adjacent tissues (n = 30) were obtained from OC patients. The stage of OC patients was determined according to the clinicopathological features. Collected tissues were frozen in liquid nitrogen for further study. This study was approved and supervised by the ethics committee of Nanjing Medical University for peer review. All patients were informed and signed written consent. The basic clinico-pathological details of the patients are show in Table 1.

**Table 1:** Clinical and pathological parameters

Clinical parameters	MNX1-AS1		*P* value
	Low expression (*n* = 14)	High expression (*n* = 16)	
Age			0.073
> 50	7	9	
≤ 50	7	7	
FIGO			0.069
I ~ II	8	7	
III ~ IV	6	9	
Tumor Size			0.036
> 2 cm	5	11	
≤ 2 cm	9	5	
Lymph nodes status			0.022
Negative	10	6	
Positve	4	10	

### Cell culture

OC cell lines (A2780, SKOV-3, OVCAR-3, HO8910) and regular ovarian cell lines (ISOE80) were purchased from the Cell Resource Center of Shanghai Academy of Sciences (Shanghai, China). Passage numbers of all cell lines were maintained between 6 and 7 for all cell lines. These cells were cultured with Roswell Park Memorial Institute (RPMI) 1640 medium or Dulbecco’s Modified Eagle medium (DMEM) (Thermo-Scientific, MA, USA), which contained 10% fetal bovine serum (FBS, Thermo-Scientific, MA, USA) and 1% penicillin-streptomycin (MP Biomedicals, CA, USA). The cells were maintained in an incubator at 37 °C and 5% CO_2_.

### Cell transfection

To obtain stable knockdown cell lines, lncRNA MNX1-AS1 shRNA lentiviruses and control shRNA lentiviruses were inserted into the pLVX-tdTomato-Puro vector (GenePharma, Shanghai, China) and transduced into SKOV-3 cells and OVCAR-3 cells, respectively.

To obtain transiently transfected cell lines, miR-744-5p mimics, negative control (NC) mimics, miR-744-5p inhibitor and NC inhibitor (RiboBio, Guangzhou, China) were transfected into SKOV-3 cells and OVCAR-3 cells respectively by using lipofectamine 3000 (Thermo-Scientific, MA, USA), miR-744-5p inhibitor and SOX12 shRNA (Ribobio, Guangzhou, China) were transfected into SKOV-3 cells and OVCAR-3 cells simultaneously. After 48-h transfection, cells were collected for subsequent experiments. The sequences are shown in Table 2.

**Table 2:** Sequences used for cell transfection

Genes	Sequences (5′-3′)
miR-744-5p mimics	UGCGGGGCUAGGGCUAACAGCA
NC mimics	UACUGAGAGACAUAAGUUGGUC
miR-744-5p inhibitor	AGGGCUAACAGCAGUCUUACU
NC inhibitor	CAGUACUUUUGUGUAGUACAA
sh-NC	CCTAAGGTTAAGTCGCCCTCG
sh-SOX12	GCTGCTTCACAGGATGAAA
sh-NC	CCTAAGGTTAAGTCGCCCTCG
sh-MNX1-AS1	GGUCGAACCUUAUCUGCUA

### Real-time quantitative polymerase chain reaction (RT-qPCR)

Total RNA was isolated from tissues and cells using Trizol reagent (Thermo-Scientific, MA, USA) according to the manufacture’s protocol. Then, RNA was reverse transcribed to cDNA by using PrimeScript RT Master Mix (Takara, Japan). Subsequently, RT-qPCR was performed using SYBR Green PCR Kit (Vazyme, Nanjing, China) through Applied Biosystems 7300 Real-time PCR system (Applied Biosystems, USA). The parameters for PCR amplification were as follows: 5 min at 95 °C, followed by 35 cycles of 60 s at 95 °C, 60 s at 58 °C and 90 s at 72 °C, 8 min at 72 °C. U6 and GAPDH were used to normalize the relative expression. Primer sequences used for RT-PCR are shown in Table 3.

**Table 3:** Primer sequences used for RT-qPCR

Genes	Primer sequences (5′-3′)
MNX1-AS1	
Forward	CACCAACGGGGAGTGGATAC
Reverse	CTCCAGGGACCAACCAAGTC
miR-744-5p	
Forward	GTGCGGGGCTAGGGCTA
Reverse	AGTGCAGGGTCCGAGGTATT
SOX12	
Forward	CTGGAGTGGTGGGATTGGTC
Reverse	GGGTGTCAGAGGGACAAAGG
U6	
Forward	CTCGCTTCGGCAGCACA
Reverse	AACGCTTCACGAATTTGCGT
GAPDH	
Forward	AGTCCACTGGCGTCTTCA
Reverse	GAGTCCTTCCACGATACCAA

### Cell viability assay

CCK8 assay was performed to detect cell viability. After transfection or transduction, cells were inoculated in 96-well plates with a density of 10^3^ cells and a volume of 100 μl. Then the cells were cultured for 1 h and mixed with 10 μl CCK8 regent (Dojindo, Kumamoto, Japan) for 2 h. The optical density was measured at 450 nm by utilizing Bio-EL340 automatic microplate reader (Tek Instruments, Hopkinton, USA).

### Colony formation assay

Cells were inoculated into a 6-well plate at the density of 500 cells/well. After 14 days of culture, the cells were fixed by methanol. Next, the colonies were stained with 0.1% crystal violet solution (Beyotime, Shanghai, China) after washing with PBS 2 times. After the PBS cleaning, the colonies were observed using a microscope (Nikon, Tokyo, Japan) and counted using ImageJ software (ImageJ v.1.48, http://imagej.nih.gov/ij/).

### Cell cycle analysis

Cells were collected and fixed with 75% methanol for 4 h at 4 °C. The cells were incubated with an RNA enzyme-containing iodide (PI, Sigma-Aldrich, MI, USA) after centrifuging and the supernatant removed. Then, the cells were washed with PBS three times, and the cell cycle was detected using FACS flow cytometry (Leica, Wetzlar, Germany).

### Cell apoptosis analysis

Transfected cells were collected and passed through 100 mesh sieves. Then the cells were incubated with Annexin V and PI solution for 15 min in darkness. Finally, the labeled cells were analyzed using FACS flow cytometry (Leica, Wetzlar, Germany).

### Wound-healing assay

Wound-healing assays detected cell migration. The cells were inoculated into 6-well plates. When cells were grown in a single layer, a 10 μl sterile pipette tip was used to scratch cells. Detached cells were removed by using a serum-free medium washed twice. Wounded areas were observed and imaged by microscopy (Nikon, Tokyo, Japan) after incubating the cell for 24 h.

### Transwell assay

Cell migration and cell invasion were evaluated by using a transwell chamber. First, the invasion assay pre-coated the transwell chamber membrane with the Matrigel (Franklin Lakes, NJ, USA). Briefly, the transfected cells were seeded into the top compartment of the chamber with the basal medium. Next, the bottom compartment of the chamber was supplemented with the medium containing 10% FBS. After incubated 48-h, the cells in the bottom compartment were fixed with methanol and stained with 0.1% crystal violet for 30 min at 37 °C. Finally, the cells were counted using ImageJ software (ImageJ v.1.48, http://imagej.nih.gov/ij/) and photographed using a microscope (Nikon, Tokyo, Japan).

### Western blot

Transfected cells were collected and extracted protein by using RIPA lysis buffer. Protein was separated by SDS-acrylamide gel and transferred to polyvinylidene difluoride membrane (PVDF, Millipore, MA, USA). Following, membranes were incubated with 5% non-fat milk for 2 h at room temperature to block non-specific antigen and then incubated with primary antibodies at 4 °C overnight. After PBST was washed three times, membranes were incubated with HRP-conjugated secondary antibody at room temperature for 2 h. Protein bands were visualized using an enhanced chemiluminescence kit (Vazyme, Nanjing, China) after PBST was washed three times. GAPDH was used as the internal control for relative protein expression. Image J software (ImageJ v.1.48, http://imagej.nih.gov/ij/) was used for quantitative analysis.

Primary antibody information is as follows: Cyclin D1 (ab16663, 1:200), p21 (ab109520, 1:2000), Bax (ab32503, 1:1000), Bcl-2 (ab182858, 1:2000), Cleaved-caspase-3 (ab32042, 1:500), Cleaved-caspase-9 (ab2324, 1:200), Cox-2 (ab179800, 1:1000), MMP-2 (ab92536, 1:1000), MMP-9 (ab76003, 1:1000), SOX12 (ab54371, 1:1000), GAPDH (ab181602, 1:10000). The secondary antibody is Goat anti-Rabbit HRP antibody (ab6721, 1:5000) and Goat anti-Mouse HRP antibody (ab205719, 1:10000). All antibody were purchased from Abcam (MA, USA).

### Nucleoplasm isolation assay

Nucleoplasm isolation assay was performed by using Nucleus-Cytoplasmin-Cytoplasmic Membrane Preparation Kit (Beijing Apply Gene Technology Co., China) according to the manufacturer’s instructions. Briefly, every 10^7^ cells were added with 500 μl CER reagent; then, the mixture was bathed for 2 min in ice and homogenized 20 times. Following, the cells in the mixture were lysed by centrifugation. The cytoplasmic was in the supernatant, and the nucleus was in the tube bottom.

### Luciferase reporter assay

LncRNA MNX1-AS1-miR-744-5p binding sites and miR-744-5p-SOX12 binding sites were identified on the bioinformatics prediction website StarBase v3.0 (http://starbase.sysu.edu.cn/). MNX1-AS1 containing miR-744-5p binding sequence was inserted into the pLVX-IRES-Puro vector (MNX1-AS1 WT). The sequence of MNX1-AS1 binding with miR-744-5p was mutated and inserted into the pLVX-IRES-Puro vector (MNX1-AS1 Mut) to confirm the specific binding. Similarly, the SOX12 3’UTR sequence containing miR-744-5p binding sequence was inserted into the luciferase reporter vector (SOX12 WT). To assess the binding specificity, the sequences of SOX12 3’UTR interacted with miR-744-5p were mutated and inserted into the equivalent luciferase reporter vector (SOX12 Mut). SKOV-3 and OVCAR-3 cells of 10^5^ were cultured in 24-well plates. Each well was transfected with a 1 μg luciferase reporter vector, 1 μg of the β-galactosidase plasmid (internal control), and 100 pM of miR-744-5p mimics or NC mimics using Lipofectamine 3000 (Thermo-Scientific, MA, USA). After 48-h transfected, luciferase activities were measured using a luciferase assay kit (Promega, WI, USA).

### Statistical analysis

All data were collected from three independent experiments and presented as the mean ± Standard deviation (SD). Results were analyzed with one-way ANOVA and student t-tests using GraphPad Prism 7.0 (GraphPad Inc., San Diego, CA, USA). *P*-value < 0.05 was considered statistically significant.

## Results

### The expression of MNX1-AS1 is upregulated in OC tissues and cells

**Fig. 1:**
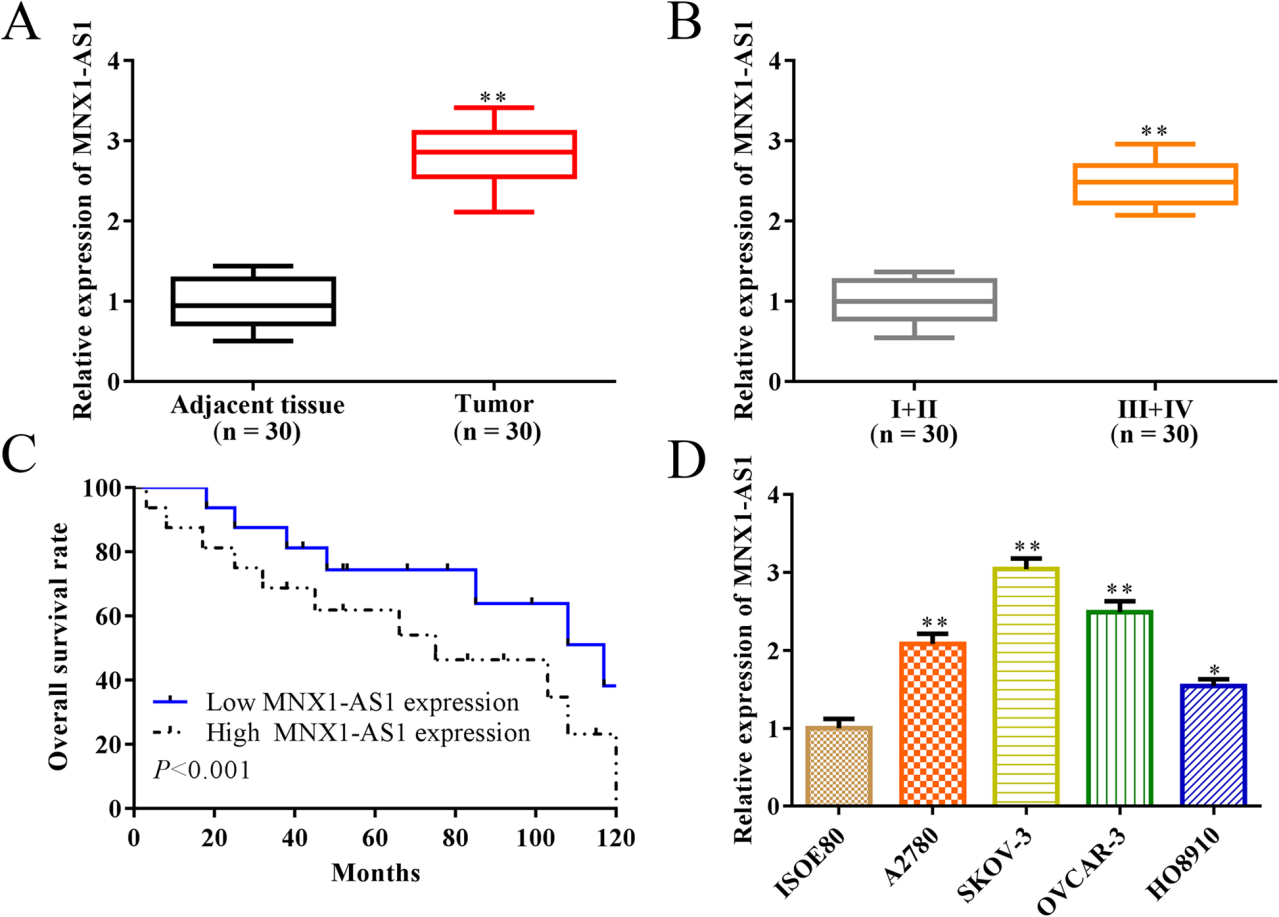
The expression of MNX1-AS1 is upregulated in OC tumor tissues and cells. **A** RT-qPCR detected the expression of MNX1-AS1 in OC tumor tissues (*n* = 30) and adjacent tissue (n = 30). **B** RT-qPCR detected the expression of MNX1-AS1 in OC tumor tissues from early-stage (I and II stage, n = 30) patients and advanced stage (III and IV stage, n = 30) patients. **C** The overall survival rates of OC patients were compared in MNX1-AS1 high expression and low expression groups. **D** RT-qPCR detected the expression of MNX1-AS1 in OC cells and normal ovarian cells. ^*^P<0.05, ^**^P<0.01, the difference comparison compared to regular group or early-stage tumor tissues. Error bars were represented the mean ± SD of triplicate experiments

To detect the roles of MNX1-AS1 in the development of OC, we collected the tumor tissues and non-tumor tissues from OC patients. Then, we detected the expression of MNX1-AS1, and the results showed that MNX1-AS1 expression was increased in OC tumor tissues compared with non-tumor tissues (Fig. 1A). Furthermore, we divided OC patients into early-stage (I and II stage) and advanced stage (III and IV stage) according to the clinical features. As a result, we detected the expression of MNX1-AS1 in tumor tissues. Furthermore, the results showed that MNX1-AS1 levels were elevated in tumor tissues from advanced stage OC patients than early-stage patients (Fig. 1B).

Furthermore, Kaplan-Meier survival analysis indicated that the high expression of MNX1-AS1 was associated with a low overall survival rate in OC patients (Fig. 1C). Similarly, we confirmed that the expression of MNX1-AS1 was upregulated in OC cells (A2780, SKOV-3, OVCAR-3 and HO8910) than the normal ovarian cells (ISOE80), especially in SKOV-3 and OVCAR-3 cells (Fig. 1D); therefore, SKOV-3 and OVCAR-3 cells were used in subsequent experiments. These results indicated that MNX1-AS1 is involved in OC development and is associated with a poor prognosis.

### Knockdown of MNX1-AS1 inhibits OC cell proliferation, blocks cell cycle, and promotes cell apoptosis

**Fig. 2:**
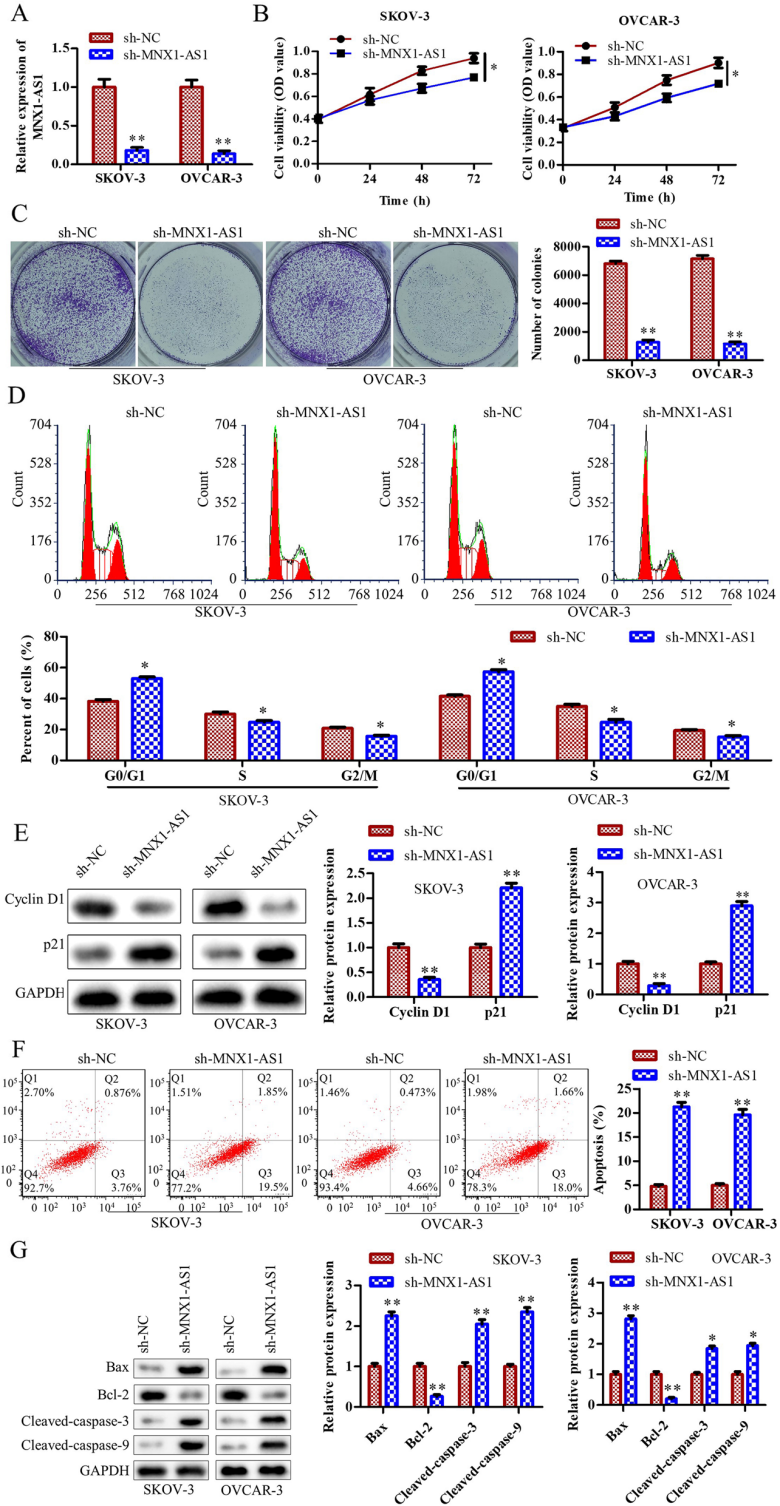
Knockdown of MNX1-AS1 inhibits OC cell proliferation, blocks cell cycle and promotes cell apoptosis. MNX1-AS1 shRNA lentivirus and NC shRNA lentivirus were transfected into SKOV-3 and OVCAR-3 cells, respectively. **A** RT-qPCR detected the expression of MNX1-AS1. **B** Cell viability was detected by CCK8 assay. **C** Cell proliferation was detected by colony formation assay. **D** Cell cycle was detected by flow cytometry. **E** Western blot was used to detect the expression of cell cycle-related genes. **F** Flow cytometry detected cell apoptosis. **G** The protein levels of apoptosis-related genes were detected by western blot. ^*^P<0.05, ^**^P<0.01, the difference comparison was compared with sh-NC transfected cells. Error bars were represented the mean ± SD in three independent repetitions

To determine the functions of MNX1-AS1 in OC, we transfected MNX1-AS shRNA lentivirus in SKOV-3 and OVCAR-3 cells. The knockdown efficiency of MNX1-AS1 was shown in Fig. 2A; the result revealed that the transfection was successful. CCK8 assay and colony formation assay were used to detect cell proliferation, and the results showed that knockdown of MNX1-AS inhibited cell viability and cell proliferation in SKOV-3 and OVCAR-3 cells (Fig. 2B and C). Subsequently, flow cytometry was used to detect the cell cycle. The results displayed that knockdown of MNX1-AS1 increased the G0/G1 phase ratio and decreased the ratio of S phase and G2/M stages in SKOV-3 and OVCAR-3 cells (Fig. 2D). Consistently, western blot results showed that knockdown of MNX1-AS1 downregulated Cyclin D1 and upregulated the expression of p21 in SKOV-3 and OVCAR-3 cells (Fig. 2E). In addition, we detected the cell apoptosis by utilizing flow cytometry and found that the cell apoptosis ratio was enhanced in sh-MNX1-AS1 cells than sh-NC cells (Fig. 2F). Consistent with this data, the expression of Bax, Cleaved-caspase-3 and Cleaved-caspase-9 were increased. Meanwhile, the expression of Bcl-2 was decreased in sh-MNX1-AS1 cells compared with sh-NC cells (Fig. 2G). These results demonstrated that knockdown of MNX1-AS1 inhibited OC cell proliferation, blocked cell cycle, and promoted cell apoptosis.

### Knockdown of MNX1-AS1 inhibits OC cell migration and invasion

**Fig. 3:**
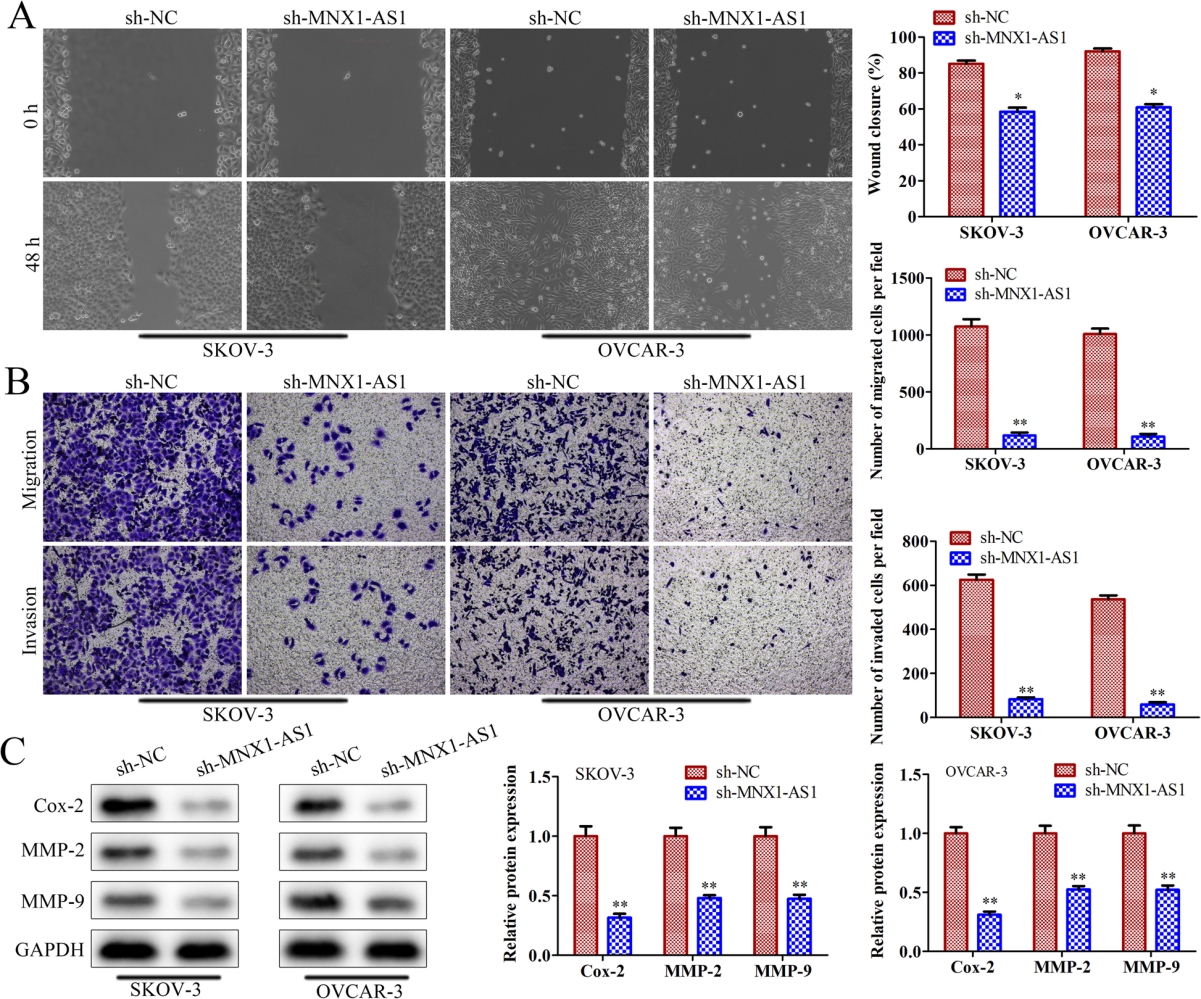
Knockdown of MNX1-AS1 inhibits OC cell migration and invasion. MNX1-AS1 shRNA lentivirus and NC shRNA lentivirus were transfected into SKOV-3 and OVCAR-3 cells, respectively. **A** Cell migration was detected by wound-healing assay. **B** Transwell assay was used to detect cell migration and invasion. **C** Western blot measured protein levels of cell migration and invasion-related genes. ^*^P<0.05, ^**^P<0.01, the difference comparison was compared with sh-NC transfected cells. Error bars were represented the mean ± SD in three independent repetitions

Further, we detected the roles of MNX1-AS1 in OC cell migration and invasion. Wound-healing assay results showed that knockdown of MNX1-AS1 inhibited cell migration in SKOV-3 and OVCAR-3 cells (Fig. 3A). Transwell assay results showed that downregulation of MNX1-AS1 expression inhibited cell migration and invasion in SKOV-3 and OVCAR-3 cells (Fig. 3B). Similarly, western blot results demonstrated that knockdown of MNX1-AS1 decreased the protein expression of migration and invasion-related genes, including Cox-2, MMP-2 and MMP-9 in SKOV-3 and OVCAR-3 cells (Fig. 3C). These results indicated that knockdown of MNX1-AS1 inhibited OC cell migration and invasion.

### MNX1-AS1 binds to miR-744-5p and inhibits its expression

**Fig. 4:**
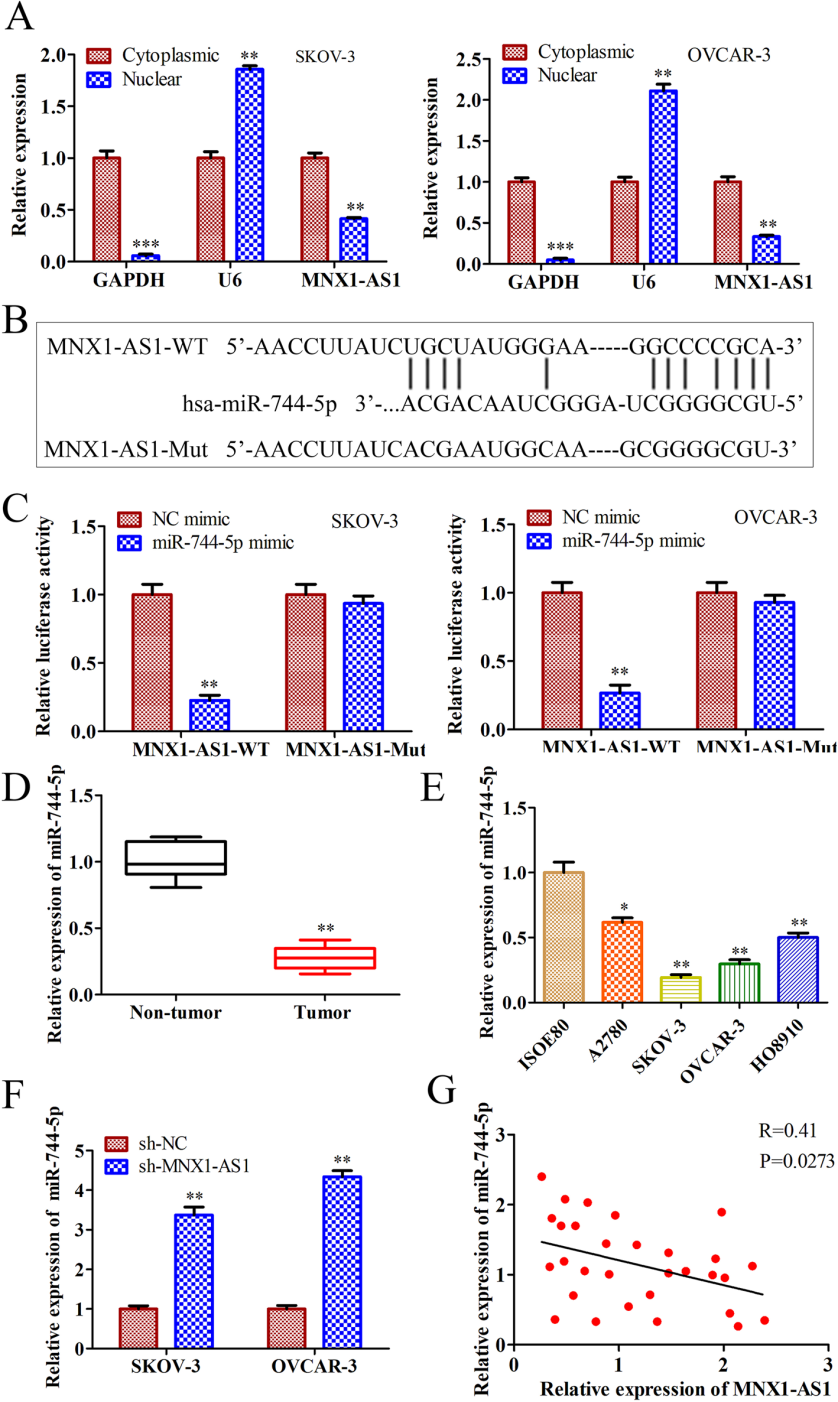
MNX1-AS1 binds to miR-744-5p and inhibits its expression. **A** RT-qPCR detected MNX1-AS1 expression in the cytoplasm and nucleus of OC cells. **B** The predicted binding sequence of miR-744-3p and MNX1-AS1, and the mutated binding sequence of MNX1-AS1. **C** Luciferase activity was detected in SKOV-3 and OVCAR-3 cells co-transfected with MNX1-AS1 WT or MNX1-AS1 Mut vector and NC mimics or miR-744-5p mimics. **D** RT-qPCR detected the expression of miR-744-5p in OC non-tumor and tumor tissues. **E** RT-qPCR detected the expression of miR-744-5p in OC cells and normal ovarian cells. **F** RT-qPCR detected the expression of miR-744-5p in SKOV-3 and OVCAR-3 cells transfected with sh-MNX1-AS1 or sh-NC. **G** Correlation analysis of MNX1-AS1 and miR-744-5p expression levels in OC tumor tissues. ^**^P<0.01,^***^P<0.001, the difference comparison was compared with control group cells and tissues. Error bars were represented the mean ± SD of triplicate experiments

To further explore the underlying mechanism of MNX1-AS1 in OC, we divided cytoplasm and nucleus from SKOV-3 and OVCAR-3 cells and detected the expression of MNX1-AS1 through RT-qPCR. The results showed that MNX1-AS1 expression was higher in the cytoplasm than in the nucleus (Fig. 4A). Increasing studies have reported that lncRNAs are located in the cytoplasm and exert roles through sponging bind with miRNA [25]. Therefore, we predicted the potential miRNA that may interact with MNX1-AS1 using Starbase v3.0 and found miR-744-5p had a potential binding sequence of MNX1-AS1. The potential binding sequence is shown in Fig. 4B. To confirm the binding of miR-744-5p to MNX1-AS1, a luciferase reporter assay was performed. The results showed that the luciferase activity was dramatically reduced in miR-744-5p mimics MNX1-AS1 WT co-transfected cells compared with NC mimics and MNX1-AS1 WT co-transfected cells. At the same time, the luciferase activity had no significant changes in MNX1-AS1 Mut transfected cells (Fig. 4C).

Additionally, we detected the expression of miR-744-5p in OC tumor tissues and cells. The RT-qPCR results displayed that the miR-744-5p level was decreased in OC tumor tissues and cells (Fig. 4D and E). Further, we detected the effect of MNX1-AS1 on miR-744-5p expression through RT-qPCR. According to the results, we found that the expression of miR-744-5p was increased dramatically in sh-MNX1-AS1 cells than in sh-NC cells (Fig. 4F). Furthermore, we confirmed a negative correlation between the expression of MNX1-AS1 and miR-744-5p in OC tissues (Fig. 4G). These results demonstrated that miR-744-5p was a target of MNX1-AS1 in OC, and MNX1-AS1 inhibited the expression.

### Overexpression of miR-744-5p inhibits OC cell proliferation, migration, invasion and promotes cell apoptosis

**Fig. 5:**
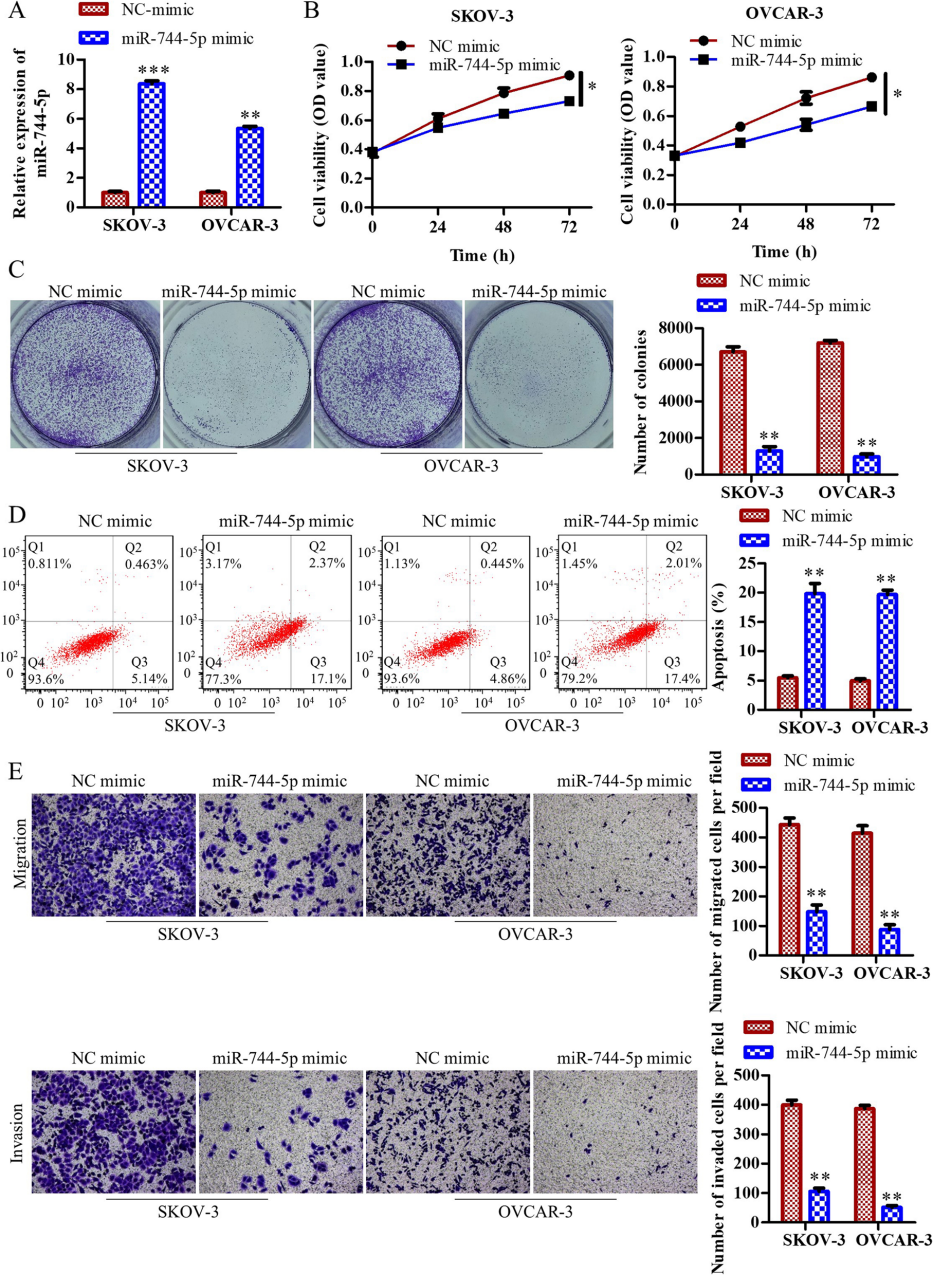
Overexpression of miR-744-5p inhibits OC cell proliferation, migration, invasion and promotes cell apoptosis. SKOV-3 and OVCAR-3 cells transfected miR-744-5p mimics and NC mimics, respectively. **A** RT-qPCR detected the efficiency of miR-744-5p overexpression. **B** Cell viability was detected by CCK8 assay. **C** Colony formation assay was used to detect cell proliferation. **D** Cell apoptosis was detected by flow cytometry. **E** Transwell assay was used to detect cell migration and invasion. ^*^P<0.05, ^**^P<0.01,^***^P<0.001, the difference comparison was compared with NC mimics transfected cells. Error bars were represented the mean ± SD in three independent repetitions

To determine the regulatory effect of miR-744-5p in OC, we transfected miR-744-5p mimics into SKOV-3 and OVCAR-3 cells. NC mimics were transfected as a negative control. The overexpression of miR-744-5p was confirmed by RT-qPCR (Fig. 5A). CCK8 assay and colony formation assay results showed that overexpression of miR-744-5p decreased cell viability and cell proliferation in SKOV-3 and OVCAR-3 cells (Fig. 5B and C). Flow cytometry results showed that cell apoptosis ratio was enhanced in miR-744-5p mimics transfected cells than control cells (Fig. 5D). Additionally, transwell assay results showed that miR-744-5p overexpression inhibited cell migration and cell invasion in SKOV-3 and OVCAR-3 cells (Fig. 5E). These results suggested that miR-744-5p inhibited cell proliferation, migration, invasion and promoted cell apoptosis in OC.

### SOX12 is a target gene of miR-744-5p

**Fig. 6:**
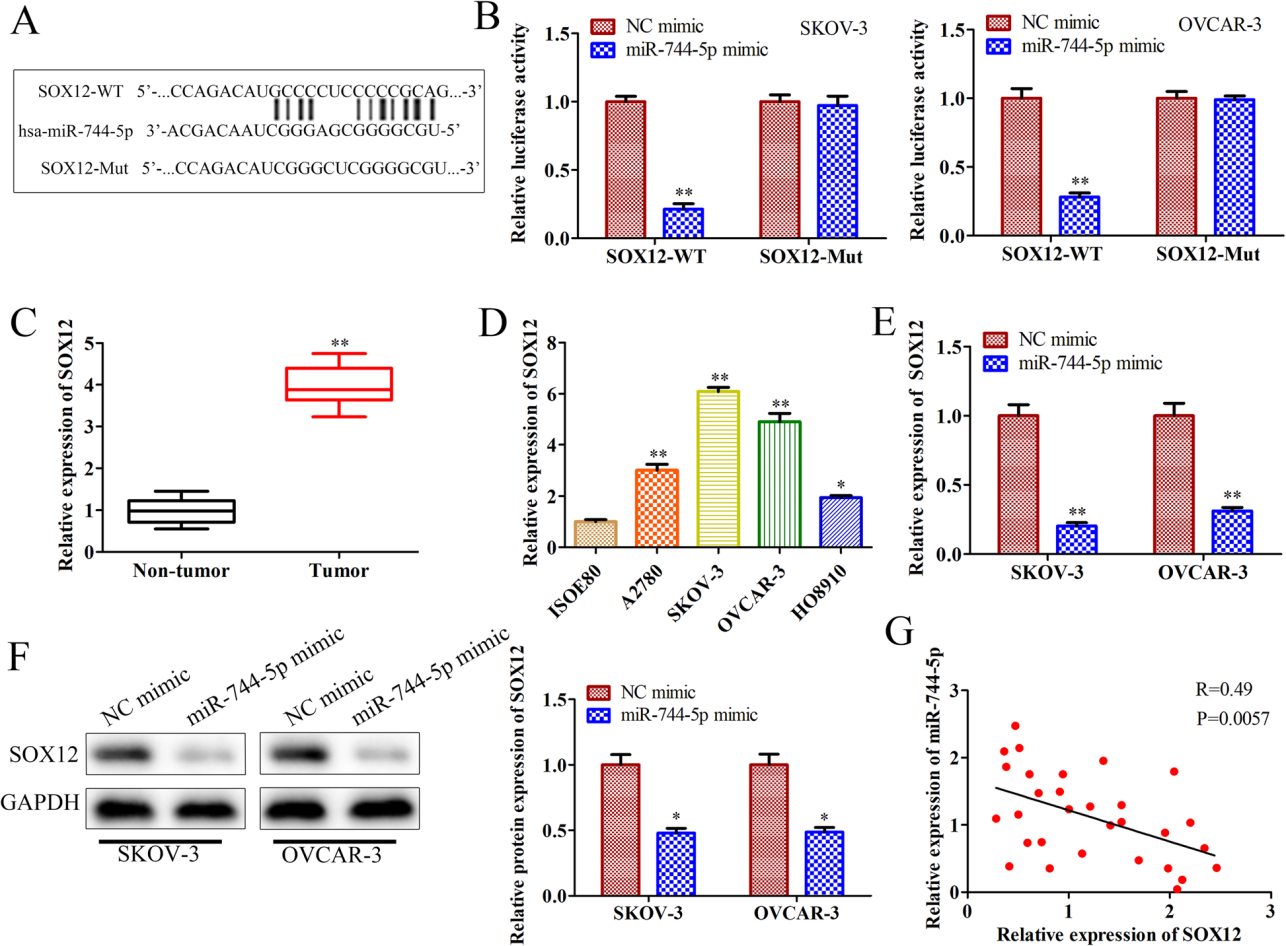
SOX12 is a target gene of miR-744-5p. **A** The predicted binding sequence of miR-744-5p and SOX12 3’UTR, and the mutated sequence of SOX12. **B** Luciferase activity was detected in SKOV-3 and OVCAR-3 cells co-transfected with SOX12 WT or SOX12 Mut vector and NC mimics or miR-744-5p mimics. **C** RT-qPCR detected the expression of SOX12 in OC tumor tissues and non-tumor tissues. **D** RT-qPCR detected the expression of SOX12 in OC cells and normal ovarian cells. NC mimics and miR-744-5p mimics were transfected into SKOV-3 and OVCAR-3 cells, respectively. The expression of SOX12 was detected by RT-qPCR (**E**) and western blot (**F**). **G** Correlation analysis of SOX12 and miR-744-5p expression levels in OC tumor tissues. ^*^P<0.05, ^**^P<0.01, the difference comparison was compared with control group cells and tissues. Error bars were represented the mean ± SD in three independent repetitions

Further, we predicted the target gene of miR-744-5p using Starbase v3.0 and found that SOX12 is the target gene of miR-744-5p. First, the binding sequence of miR-744-5p and SOX12 was shown in Fig. 6A. Then, a luciferase assay was used to verify the binding of SOX12 to miR-744-5p. The results showed that luciferase activity was significantly decreased in miR-744-5p mimics and SOX12 WT transfected cells compared with NC mimics and SOX12 WT transfected cells. In contrast, the luciferase activity had no change in SOX12 Mut transfected cells (Fig. 6B). Then, we detected SOX12 expression in OC tumor tissues and cells by RT-qPCR. The results showed that the expression of SOX12 was upregulated in OC tumor tissues and cells compared with non-tumor tissues and cells (Fig. 6C and D).

Additionally, we detected the expression of SOX12 in miR-744-5p mimics transfected cells through RT-qPCR and western blot. These results indicated that mRNA and protein expression levels of SOX12 were lower in mir-744-5p-overexpressing cells than in control cells. (Fig. 6E and F). Moreover, we confirmed a negative correlation between the expression of SOX12 and miR-744-5p in OC tumor tissues (Fig. 6G). These results demonstrated that SOX12 is a target gene of miR-744-5p, and miR-744-5p negatively regulated the expression of SOX12 in OC.

### The function of MNX1-AS1/ miR-744-5p/ SOX12 axis in OC

**Fig. 7:**
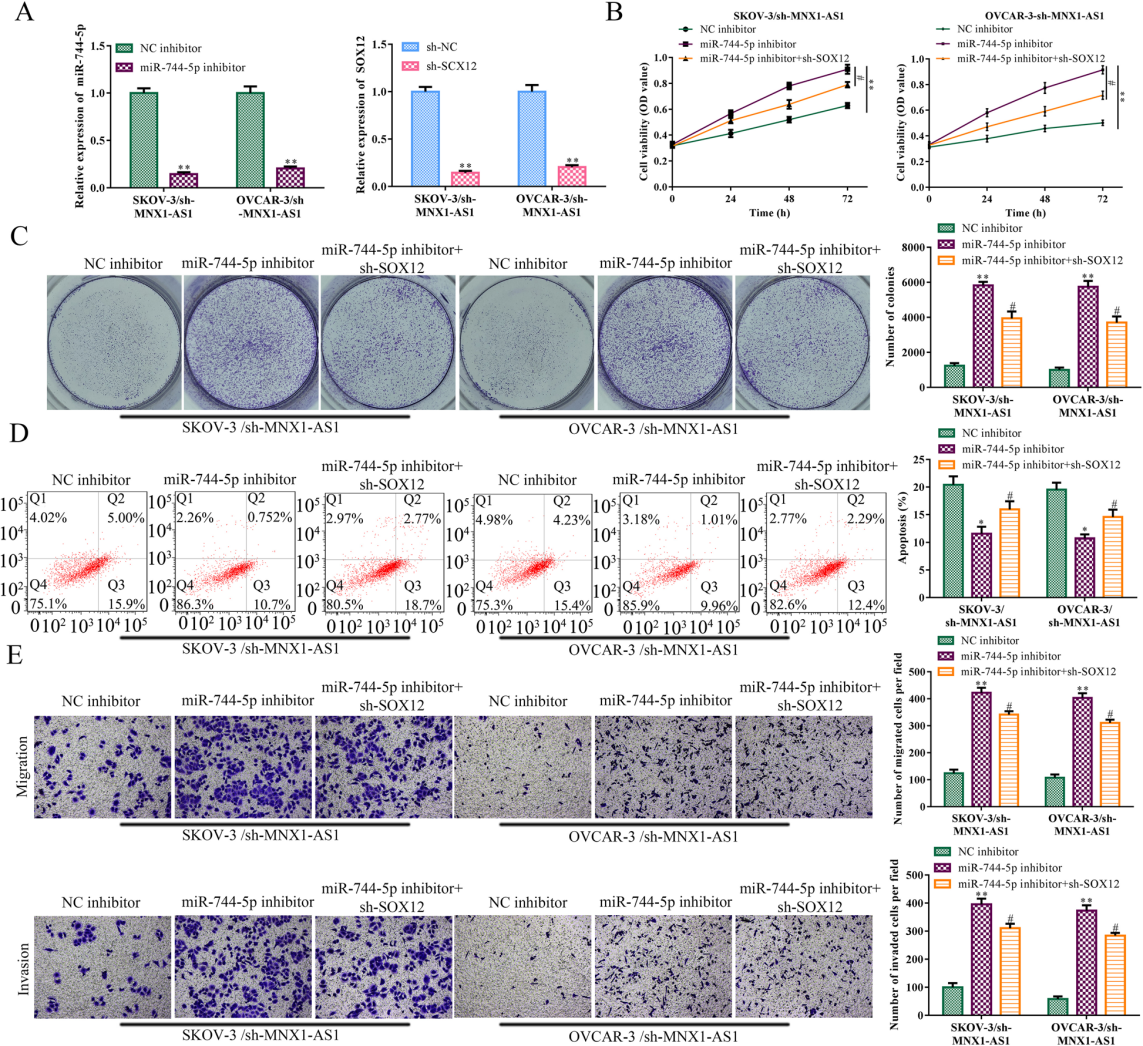
The function of MNX1-AS1/ miR-744-5p/ SOX12 axis in OC. MNX1-AS1 shRNA lentivirus was transfected into SKOV-3 and OVCAR-3 cells, respectively, to construct MNX1-AS1 knockdown stable strain. NC inhibitor, miR-744-5p inhibitor, NC shRNA and SOX12 shRNA were transfected into SKOV-3/sh-MNX1-AS1 and OVCAR-3/sh-MNX1-AS1 cells, respectively. The knockdown efficiency of miR-744-5p and SOX12 was detected by RT-qPCR (**A**). MiR-744-5p inhibitor and SOX12 shRNA were transfected into SKOV-3/sh-MNX1-AS1 and OVCAR-3/sh-MNX1-AS1 cells simultaneously. **B** Cell viability was detected by CCK8 assay. **C** Colony formation assay was used to detect cell proliferation. **D** Cell apoptosis was detected by flow cytometry. **E** Transwell assay was used to detect cell migration and invasion. ^*^P<0.05, ^**^P<0.01, the difference comparison was compared with NC inhibitor or NC shRNA transfected cells. ^***#***^*P*<0.05, the difference comparison was compared with miR-744-5p inhibitor transfected cells. Error bars were represented the mean ± SD in three independent repetitions

To further verify whether MNX1-AS1 affects the process of OC via miR-744-5p/SOX12 axis, we transfected miR-744-5p inhibitors and SOX12 shRNA into MNX1-AS1 knockdown cells separately or simultaneously. RT-qPCR detected the knockdown efficiency of miR-744-5p inhibitors and SOX12 shRNA, and results were shown in Fig. 7A. CCK8 assay and colony formation assay results confirmed that knockdown of miR-744-5p increased cell viability and cell proliferation in SKOV-3/sh-MNX1-AS1 and OVCAR-3/sh-MNX1-AS1 cells. However, the increase of cell viability and cell proliferation were attenuated by the knockdown of SOX12 (Fig. 7B and C). Flow cytometry assay results showed that the downregulation of miR-744-5p decreased cell apoptosis, which was reversed by SOX12 knockdown in SKOV-3/sh-MNX1-AS1 OVCAR-3/sh-MNX1-AS1 cells (Fig. 7D). Consistently, knockdown of miR-744-5p promoted cell migration and invasion in SKOV-3/sh-MNX1-AS1 and OVCAR-3/sh-MNX1-AS1 cells. The promotion role of cell migration and invasion was attenuated by decreasing the expression of SOX12 (Fig. 7E). These results clarified that MNX1-AS1 promoted cell proliferation, migration, invasion and inhibited cell apoptosis via miR-744-5p/SOX12 pathway in OC.

## Discussion

More and more studies have shown that aberrant expression of LncRNA is closely associated with OC occurrence and development, and LncRNAs are expected to be new markers for OC diagnosis and treatment [26].

MNX1-AS1 has shown to be an oncogene in multiple cancers. For instance, overexpression of MNX1-AS1significantly promoted the proliferation, migration, invasion and angiogenesis of intrahepatic cholangiocarcinoma cells in vitro. And tumor growth and metastasis in vivo [14]. MNX1-AS1 is significantly upregulated in cervical cancer and lung cancer tissues and cell lines [15, 16]. Similarly, according to our result, MNX1-AS1 is upregulated in OC tissues and cells. In addition, we found that the high expression of MNX1-AS1 was associated with a low overall survival rate in OC patients. Further, knockdown of MNX1-AS1 inhibits OC cell proliferation, blocks cell cycle, and promotes cell apoptosis.

Previous studies have reported that lncRNAs regulate biological function at epigenetic, transcriptional and post-transcriptional levels or directly regulate protein activity [27]. In addition, lncRNAs often function as ceRNAs binding to miRNA when located in the cytoplasm, regulating multiple target gene expressions [28]. Hence, we detected the expression of MNX1-AS1 in the cytoplasm and nucleus of OC cells. The results displayed that MNX1-AS1 is mainly located in the cytoplasm of OC cells. Therefore, we explored miRNAs that target MNX1-AS1 and found miR-744-5p was a predicted miRNA with a potential binding sequence. Subsequently, we confirmed that miR-744-5p targets are binding with MNX1-AS1 through luciferase reporter assay.

Furthermore, MiR-744-5p has been shown to slow the progression of OC [21, 29]. Therefore, we speculated that MNX1-AS1 regulates the OC process through sponging to miR-744-5p. We determined that MNX1-AS1 negatively regulated miR-744-5p expression and miR-744-5p overexpression inhibited cell proliferation, migration, invasion and promoted cell apoptosis.

MiRNAs binding to the 3’UTR of the downstream target gene and regulating gene translation process then affect cancer development [30]. Based on the predicted results and RT-qPCR results, we found SOX12 is a target gene of miR-744-5p, and the expression was increased in OC tumor tissues and cells. SOX12’s promotion function has been reported in several cancers, including OC [31–33]. We confirmed that miR-744-5p negatively regulated SOX12 expression. Rescue experimental results proved that the effects of MNX1-AS1 knockdown could be reversed by downregulation of miR-744-5p, and the knockdown of SOX12 abolished such a reversible effect.

In conclusion, we demonstrated that the upregulation of MNX1-AS1 in OC tissues and cells. Moreover, we confirmed that MNX1-AS1 promoted OC development through the miR-744-5p/SOX12 axis. These results reveal a novel mechanism of MNX1-AS1 in OC, which may provide a new treatment or scanning target for OC.

However, the results of this study are limited to in vitro studies. At present, there is no evidence to show the effect of MNX1-AS1 on tumor growth or metastasis in vivo and its clinical significance in OC. Therefore, a more in-depth research is needed in the future. In addition, there is sufficient evidence that LncRNA can regulate cell phenotype by sponge effect on several different miRNAs and controlling transcription, translation or post-translational modification by binding with proteins. Similarly, each miRNA can bind and regulate several different target genes simultaneously [34–36]. Therefore, this study’s MNX1-AS1/miR744-5p/SOX12 pathway is likely to be only one of the possible pathways affecting the occurrence and development of OC.

## Supplementary Information

**Additional file 1.**

**Additional file 2.**

**Additional file 3.**

## Data Availability

All data generated or analyzed during this study are included in this published article.
