# Supplementary figures and images for: LncRNA MNX1-AS1 promotes ovarian cancer process via targeting the miR-744-5p/SOX12 axis
Source: J Ovarian Res. 2021 Nov 17;14:161. doi: 10.1186/s13048-021-00910-0 (PMC8596928; doi:10.1186/s13048-021-00910-0)

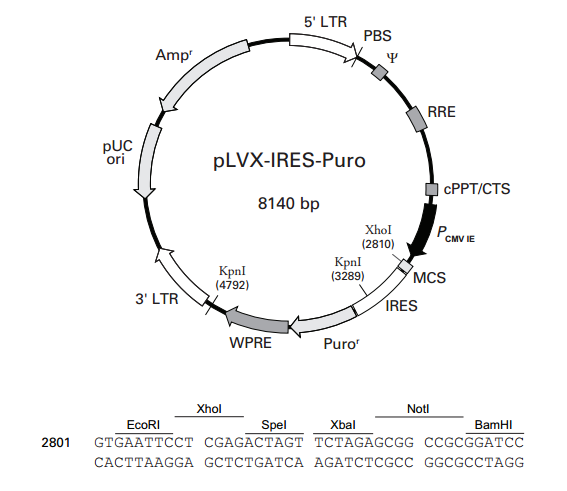

Supplement: Supplementary file 1 — Additional file 1. [file 13048_2021_910_MOESM1_ESM.png]
