# Supplementary material for: LncRNA MNX1-AS1 promotes ovarian cancer process via targeting the miR-744-5p/SOX12 axis
Source: J Ovarian Res. 2021 Nov 17;14:161. doi: 10.1186/s13048-021-00910-0 (PMC8596928; doi:10.1186/s13048-021-00910-0)

STR 基因型检测报告

样本名称：SKOV-3

检测方法：用 Axygen 的基因组抽提试剂盒提取 DNA，采用 20-STR 扩增方案扩增，在 AB13730XL 型遗传分析仪上对 STR 位点和性别基因 Amelogenin 进行检测。

检测结果：该株细胞 DNA 分亚在细施系检索中找到**完全匹配**的细施系，DSMZ 数据库显示细能名为 **SKOV-3**，细胞号对应 **161**。本次检测在该细胞系中**没有发现多等位基因。**

细胞匹配值：1.0

STR 数据库比对分析：待测细胞的 STR 位点和 Amelogenin 位点的基因分型结果与收录于 ExPASY, ATCC, DSMZ, JCRB 和 RIKEN 数据降的 2455 个细胞系 STR 数据进行比对，如果待检测细胞未收录于以上细胞库或这是自行建立的新细胞系将无法进行比对，用户需据细胞分型结果自行与其他数据库进行比对。

分型结果：

Loci	送检细胞 STR 信息			细胞库细胞 STR 信息		
	送检细胞名：PC28			细胞库细胞名：SKOV-3		
	Allele1	Allele2	Allele3	Allele1	Allele2	Allele3
CSF1PO	11	11		18	23	
D251338	18	23		14	14	
D3S1358	14	14		11	11	
D5S818	11	11		13	14	
D7S820	13	14		14	15	
D8S1179	14	15		8	11	
D13S317	8	11		12	12	
D16S539	12	12		18	23	
D18S51	16	17				
D19S433	12	12				
D21S11	30	31				
FGA	21	24				
PentaD	12	13				
PentaE	5	13				
TH01	9	9.3				
TPOX	8	11				
vWA	17	18				
D1S1656	11	17.3				
D6S1043	12	12				
D12S391	22	23				

分型图谱：

Applied Biosystems
GeneMapper Software 5

PC28

Sample File	Sample Name	Panel	SQI	OS	SQ
PC28.fsa	PC28	PowerPlex_21_v1.1			

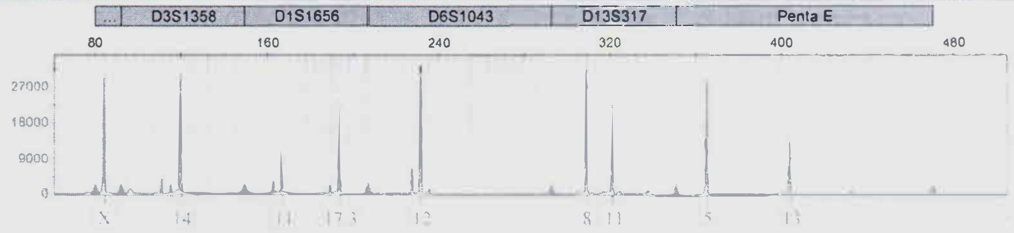

PC28.fsa PC28 PowerPlex_21_v1.1

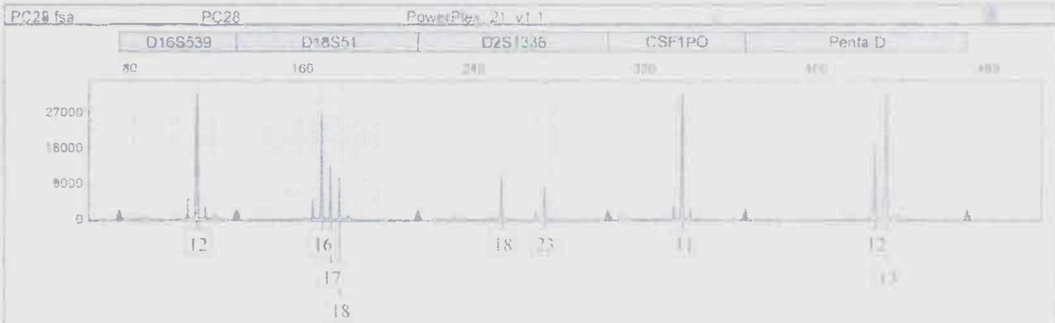

PC28.fsa PC28 PowerPlex_21_v1.1

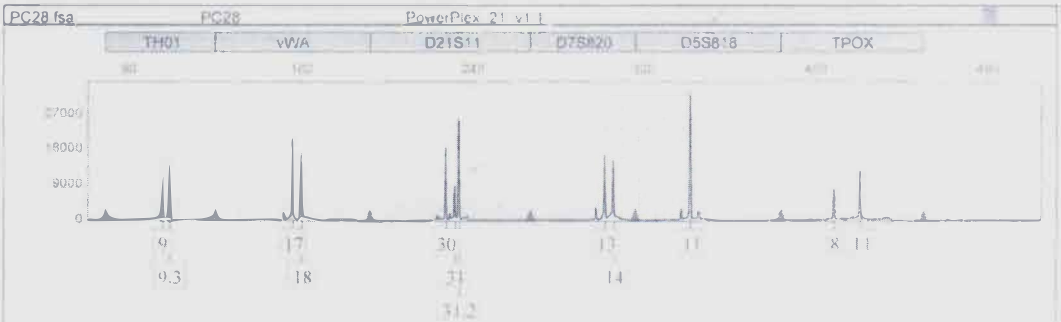

Mon Jul 25,2016 03:25PM, CST Printed by: gm

Applied Biosystems
GeneMapper Software 5

PC28

Sample File	Sample Name	Panel	SQI	OS	SQ
PC28.fsa	PC28	PowerPlex_21_v1.1		✓	

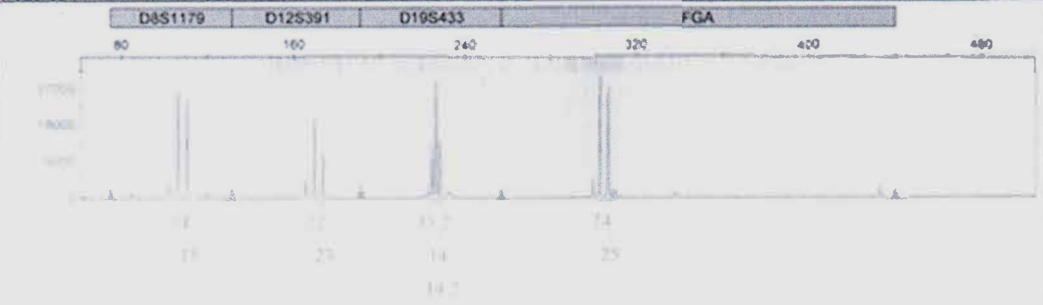

Supplement: Supplementary file 3 — Additional file 3. [file 13048_2021_910_MOESM3_ESM.pdf]
